# Hydrothermal Synthesis of CeO_2_-SnO_2_ Nanoflowers for Improving Triethylamine Gas Sensing Property

**DOI:** 10.3390/nano8121025

**Published:** 2018-12-08

**Authors:** Dongping Xue, Yan Wang, Jianliang Cao, Zhanying Zhang

**Affiliations:** 1 The Collaboration Innovation Center of Coal Safety Production of Henan Province, Jiaozuo 454000, China; xdongping1231@126.com (D.X.); caojianliang@hpu.edu.cn (J.C.); 2 School of Materials Science and Engineering, Henan Polytechnic University, Jiaozuo 454000, China

**Keywords:** CeO_2_-SnO_2_, nanostructure, hydrothermal, triethylamine, gas sensor

## Abstract

Developing the triethylamine sensor with excellent sensitivity and selectivity is important for detecting the triethylamine concentration change in the environment. In this work, flower-like CeO_2_-SnO_2_ composites with different contents of CeO_2_ were successfully synthesized by the one-step hydrothermal reaction. Some characterization methods were used to research the morphology and structure of the samples. Gas-sensing performance of the CeO_2_-SnO_2_ gas sensor was also studied and the results show that the flower-like CeO_2_-SnO_2_ composite showed an enhanced gas-sensing property to triethylamine compared to that of pure SnO_2_. The response value of the 5 wt.% CeO_2_ content composite based sensor to 200 ppm triethylamine under the optimum working temperature (310 °C) is approximately 3.8 times higher than pure SnO_2_. In addition, CeO_2_-SnO_2_ composite is also significantly more selective for triethylamine than pure SnO_2_ and has better linearity over a wide range of triethylamine concentrations. The improved gas-sensing mechanism of the composites toward triethylamine was also carefully discussed.

## 1. Introduction

Triethylamine (TEA) is a colorless, transparent oily liquid with strong ammonia odor and was widely used as an organic solvent, raw material, polymerization inhibitor, preservative, catalyst and synthetic dye [1,2]. However, TEA is also a flammable, explosive and toxic volatile organic gas that can harm human health, such as eye and skin irritation, dyspnea, headache, nausea and even death [3,4,5,6]. Therefore, it is very important to develop a method for detecting and monitoring the concentration of TEA. Up to now, gas/liquid/solid chromatography, gel chromatography, ion mobility spectrometry, electrochemical analysis and colorimetry and other methods have been explored to monitor TEA gas. However, these methods require expensive equipment and complex detection processes, which hamper their widespread use in real life [6,7,8,9,10,11,12]. Thus, it is very necessary to develop a device that is simple to manufacture and easy to detect TEA.

Metal oxide semiconductor (MOS) based gas sensors have been widely investigated in recent years because of their advantages such as simple detection, simple preparation, low cost, high sensitivity and good real-time performance [13,14,15]. SnO_2_ is a wide band gap n-type MOS material with a band gap width of 3.62 eV at room temperature. It is widely used in various fields such as photocatalysts [16,17], solar cells [18,19], lithium-ion batteries [20,21] and gas sensors [22,23,24]. As a gas sensor, SnO_2_ is one of the most widely considered gas sensitive materials due to its better gas sensitivity to various organic and toxic gases. However, we also know that the traditional SnO_2_ based gas sensors has some obvious problems of low gas response, high optimum working temperature, poor selectivity and stability [25,26,27]. Hence, researchers use another MOS doped SnO_2_ to improve its gas sensitivity. For example, Zhai et al. [28] prepared Au-loaded ZnO/SnO_2_ heterostructure, this sensor not only reduces the optimum operating temperature of SnO_2_ but also has a higher response to TEA than pure SnO_2_. Yang et al. [24] synthesized porous SnO_2_/Zn_2_SnO_4_ composites and their response to 100 ppm of TEA was about 2–3 times higher than that of pure SnO_2_. Yan et al. [29] reported a kind of porous CeO_2_-SnO_2_ nanosheets, which exhibited excellent gas sensing properties toward ethanol compared with the pure SnO_2_. These works have confirmed that another MOSs doped SnO_2_ can indeed improve gas sensing properties. As a rare earth element, Ce not only has the highest abundance of elements but also has some special characteristics such as high oxygen storage capacity, rich oxygen vacancies and low redox potential [29,30,31], these characteristics make it an ideal candidate for gas sensing materials [32,33], such as Motaung et al. [34] synthesizing CeO_2_-SnO_2_ nanoparticles with a dramatic improvement in sensitive and selective to H_2_, Dan et al. [35] prepared novel Ce-In_2_O_3_ porous nanospheres for enhancing methanol gas-sensing performance. Xu et al. [12] reported that a cataluminescence gas sensor based on LaF_3_-CeO_2_ has better sensitivity and selectivity for TEA. However, as far as we know, there are few reports about the materials of CeO_2_-SnO_2_ used to detect TEA.

Herein, we synthesized the flower-like CeO_2_-SnO_2_ composites via a facile hydrothermal method. The gas sensing performance of the flower-like CeO_2_-SnO_2_ composites based sensors were tested. The experimental results indicate that the TEA gas sensing performance of the CeO_2_-SnO_2_ composites based sensors are significantly improved by the modification of a small amount of CeO_2_ compared with pure SnO_2_, especially in terms of sensitivity and selectivity. The improvement of gas sensing properties of the composite materials is mainly due to the formation of n-n heterojunction between CeO_2_ and SnO_2_.

## 2. Results and Discussion

### 2.1. Sample Characterization

**Figure 1:**
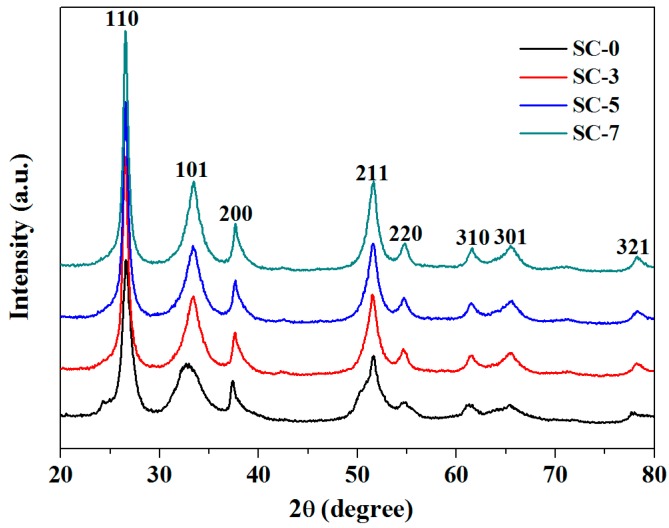
XRD patterns of the samples.

Figure 1 displays XRD patterns of the pure SnO_2_ (SC-0), 3, 5 and 7 wt.% CeO_2_ decorated SnO_2_ (SC-3, SC-5, SC-7) samples. It can be seen from Figure 1 that the XRD peaks are sharp and coincide with those of the tetragonal rutile of SnO_2_ in the space group P42/mnm with lattice constants of a = b = 4.738 Å and c = 3.187 Å (JCPDS file No. 41−1445). Apart from, all samples showing the same crystal planes of (110), (101), (200), (211), (220), (310), (301) and (321), respectively. However, no reflections characteristic of CeO_2_ was observed even at the maximum CeO_2_ content of 7 wt.%, which may be mainly due to less CeO_2_ loading. Nevertheless, one can also see from the figure that all the diffraction peak becomes sharper as the content of Ce increases. According to Debye-Scherrer principle as shown in Equation (1), where K (K = 0.89) is the Scherrer constant, D is the crystallite size, λ (λ = 0.15406 nm) is the X-ray wavelength, B is the half-height width of the diffraction peak of the measured sample, *θ* is the diffraction angle. The calculated average particle sizes of SC-0, SC-3, SC-5 and SC-7 were 11.2350, 14.0154, 14.4425, 14.633, respectively. The combined diffraction peaks became sharper, indicating that CeO_2_ was indeed loaded onto SnO_2_, since the n-n heterojunction is formed between CeO_2_ and SnO_2_ and the average particle size of the composite becomes larger as the CeO_2_ dopant increases.

(1) $$D=\frac{K\lambda }{B\cos\theta }$$

**Figure 2:**
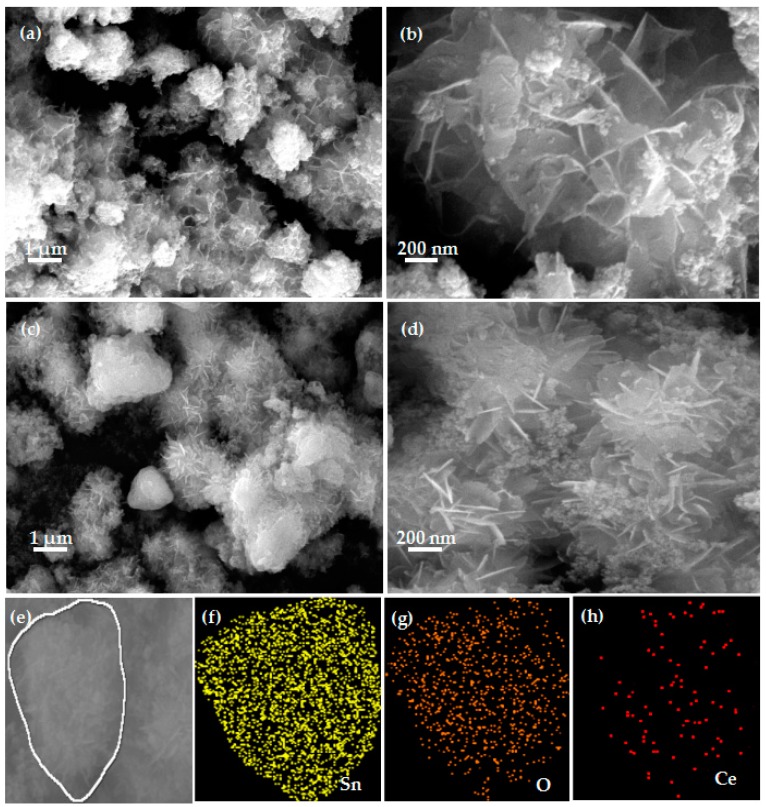
Field-emission scanning electron microscopy (FESEM) images of pure SnO_2_ (**a**,**b**) and SC-5 nanocomposite (**c**,**d**) and the EDS images (**e**-h) of the SC-5 sample.

The morphology and structure of pure SnO_2_ (Figure 2a,b) and SC-5 nanoflowers (Figure 2c,d) were observed by SEM and the presence of Ce dopant in the SC-5 nanoflowers (Figure 2e–g) was detected by EDS as illustrated in Figure 2. In Figure 2a,b, the SnO_2_ sheets are gathered together to look like a spider web and have no flower-like structure. While, in Figure 2c,d, one can be clearly seen that SC-5 has a flower-like structure with an average diameter of about 1 µm and many nanoparticles are relatively evenly dispersed on the surface of the flower-like structure. By EDS detection as shown in Figure 2e–g, it was proved that only three elements of Sn, O and Ce were found in the composite, which proved that the sample had a relatively high purity.

**Figure 3:**
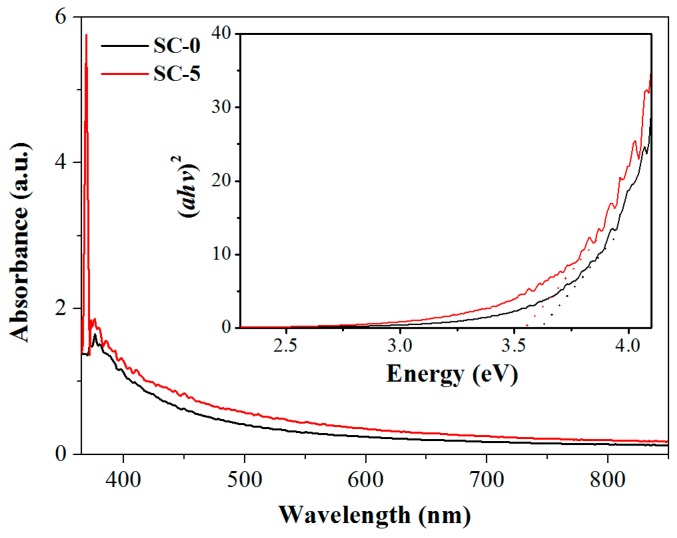
UV–vis absorption spectra of the synthesized SC-0 and SC-5 samples. The upper right corner inset is the relationship lines of (*αhv*)^2^ and *hv*.

It is well known that the presence of a heterojunction affects the band gap width of a material. Hence, in order to further illustrate the formation of n-n heterojunctions, we investigated the band gap energies of the synthesized SnO_2_ and SC-5 by UV-vis absorption spectra. As can be seen in Figure 3, the absorption peak of the red SC-5 sample moved significantly upward compared to the pure SnO_2_ curve. The relationship diagram between *(αhv)^2^* and photon energy *hv* (illustration in the upper right corner of Figure 3) is obtained according to the formula *(αhv)^2^ = A (hv − E_g_)*, where α is the absorption index, *h* is the Planck constant, *v* is the light frequency, *E_g_* is the semiconductor bandgap width, *A* is a constant associated with the material. It can be seen from the illustration that the band gap energy values of pure SnO_2_ and SC-5 are 3.64 eV and 3.56 eV, respectively. The data show that the doping of CeO_2_ narrows the band gap energy of the composite, which further proves that an n-n heterojunction is formed between composite nanomaterials.

### 2.2. Gas Sensing Performance

**Figure 4:**
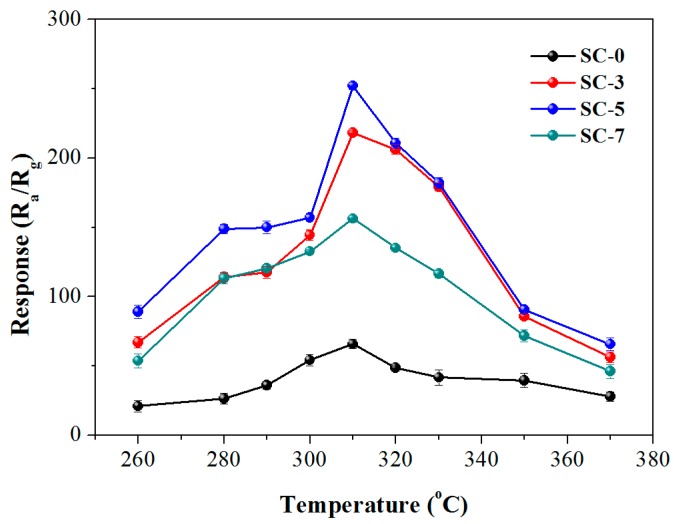
The response of the synthesized samples to 200 ppm TEA at different operating temperatures.

In order to study the gas sensing property of the as-synthesized samples to TEA, a series of examinations on the pure SnO_2_ and CeO_2_-SnO_2_ composites were performed. Because the working temperature has great impact on the response of the gas sensor, the response of four sensors to 200 ppm TEA at different temperatures was first studied, as shown in Figure 4. One can see from Figure 4 that the four curves show similar variation tendency, which is first increase and then decrease as the working temperature increases. And the response of all the sensors reached their top value at the working temperature of 310 °C. This may be due to the amount of chemisorbed oxygen ions on the surface of the sensor has reached a sufficient amount to react with TEA and the effective reaction on the surface of MOS causes an eminent change in resistance [36]. Moreover, the response of the sensors based on SC-0, SC-3, SC-5 and SC-7 are 65.77, 218.12, 252.21, 156.38, respectively. It can also be clearly seen from the Figure 4 that all composite sensors have higher response to TEA than pure SnO_2_ and the sensor based on SC-5 show higher response value than the response of the other two composite sensors. The TEA gas-sensitive properties of the SC-5 composite prepared in this work and the materials reported in other literatures [4,28,37,38,39,40,41] are shown in Table 1. Although the sensitivity of the CeO_2_-SnO_2_ sensor is higher than that of the reported results, the optimum operating temperature has not been improved very well. Therefore, we still need to do more in-depth research on reducing the working temperature, such as introducing photoexcitation equipment [42] or using p-type semiconductor doping modification [43].

**Figure 5:**
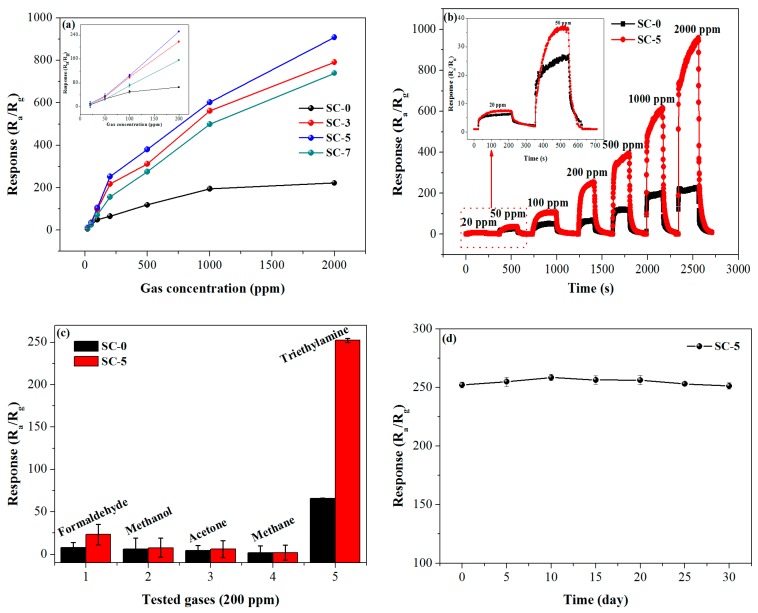
(**a**) Response of the sensors to different TEA concentrations at 310 °C (the inset shows the response curve in the range of 20–200 ppm), (**b**) dynamic response-recover curves of the sensors to different TEA concentrations at 310 °C, (**c**) responses of the SC-0 and SC-5 sensors to five gases of 200 ppm, (**d**) long-term stability measurements of the SC-5 sensor to 200 ppm TEA at 310 °C.

Sensitivity, selectivity and stability are also three important properties for evaluating sensor quality. Figure 5a exhibits the response of the four gas sensors to different TEA concentrations in the range of 20–2000 ppm at 310 °C. Obviously, the response of the four gas sensors increases with the increase of TEA concentration. It can also be seen that the response is almost linearly related to TEA concentration and the slope of the curve increase rapidly as TEA concentration increases in the concentration range of 20–200 ppm (inset of Figure 5a). Above 200 ppm, the responses increase slowly as the gas concentration increases, indicating that the adsorption of TEA by the sensor gradually becomes saturated. In addition, the SC-5 sensor exhibits higher response than that based on SC-0, SC-3 and SC-5 at different TEA concentrations. Figure 5b shows the dynamic response-recovery curves of the SC-0 and SC-5 sensors to TEA in the concentration range of 20–2000 ppm at 310 °C. As can be seen from Figure 5b that as the TEA concentration increases from 20 to 2000 ppm, the response amplitude of the two sensors gradually increases. The SC-5 composite sensor has a much higher response to the same TEA concentration than the pure SnO_2_ sensor, indicating improved gas sensitivity of the composite. Moreover, after several times of gas injection, the output voltage of the sensor in air can still return to the original value, which means that the sensor has better repeatability. Figure 5c displays the results of selective testing of the SC-0 and SC-5 sensors for five different 200 ppm reducing gases, including formaldehyde, methanol, acetone, methane and triethylamine. As can be seen in Figure 5c, the flower-like SC-5 sensor has a significantly higher response to all detected reducing gases than the response of the SC-0 sensor, further demonstrating that the SC-5 sensor has better sensitivity to reducing gases. Moreover, The SC-5 sensor responds to 200 ppm of triethylamine up to 252.2, which is 10.9, 32, 40.6 and 117.2 times higher than formaldehyde, methanol, acetone and methane, respectively, which means that the SC-5 sensor has good selectivity for triethylamine. From the perspective of the practical application of the sensor, long-term stability is also an important factor in evaluating the property of the sensor. Figure 5d displays the durable response of the SC-5 sensor to 200 ppm TEA after storing for 30 days. As shown by the curve, the response remains almost constant and remains at around 250. It can be concluded that the SC-5 sensor had better stability and can be a promising candidate for TEA sensor.

### 2.3. Gas Sensing Mechanism

**Figure 6:**
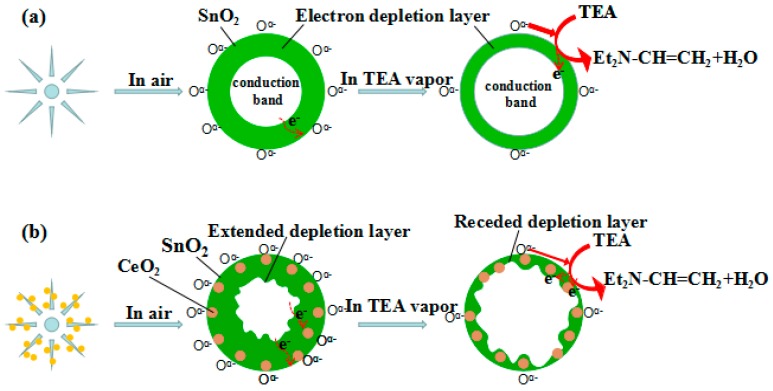
TEA sensing mechanisms diagram of (**a**) pure SnO_2_ and (**b**) CeO_2_/SnO_2_ nanostructure.

As is known, the gas sensing mechanism of n-type MOS is based on the resistance change of the sensor by the adsorption and desorption reaction of oxygen molecules on the material surface with the gas to be detected [39]. When the undoped SnO_2_ sensor is exposed to the air, as shown in Figure 6a, the oxygen molecule (O_2_) are physically adsorbed on the surface of SnO_2_ material and then convert from physisorption to chemisorption. Chemisorption oxygen molecules capture electrons from the SnO_2_ conduction band to form oxygen anions O^ɑ−^ (O^−^_2_, O^−^, O^2−^), which leads to the formation of electron depletion layer (EDL) on the surface of sensing material, the conduction channel in flower-like SnO_2_ is narrowed, the carrier concentration and conductivity are lowered and the resistance rises (R_a_). When TEA vapor is injected, the oxygen anion O^ɑ−^ formed on the surface of the SnO_2_ material reacts with the TEA and releases the trapped electrons back into the conduction band of the SnO_2_ sensing material. Consequence, the EDL becomes narrow, the conduction channel becomes wider and the conductivity of the sensor is enhanced, thus reducing the resistance of the sensor (R_g_).

The gas sensitivity of the CeO_2_-SnO_2_ sensor be improved may be mainly due to two factors. Firstly, when CeO_2_ nanoparticles are supported on SnO_2_ sheets, the electrons with a low work function flow from SnO_2_ to CeO_2_ with a higher work function due to different work functions of SnO_2_ and CeO_2_. Thus, an n-n heterojunction is formed between the junctions of SnO_2_ and CeO_2_, which further provides the surface of the SnO_2_ sheets more active sites, so that the electron depletion layer of the composite undergoes a relatively large change depending on the atmosphere of the gas as shown in Figure 6b, thereby improving the gas sensitivity of SnO_2_. When the composite sensor exposed to the air, more oxygen molecules will capture electrons from the conduction band of composite nanomaterials, which reduces the carrier concentration and the thickness of the EDL is further increased compared with the pure SnO_2_ sensor, so the resistance of the CeO_2_-SnO_2_ sensor is greatly increased. When the composite sensor is exposed to TEA gas, more oxygen anions O^ɑ−^ react with TEA and electrons are released back into the conduction band of the composite nanomaterials, concentration of free electrons in the conduction band increases and the electron depletion layer becomes thinner than the pure SnO_2_, resulting in a significant drop in sensor resistance. Thereby, the gas sensitivity of the sensor was improved. On the other hand, the electronic properties of CeO_2_ are also used to explain the sensitization mechanism [44]. Since CeO_2_ is a strong acceptor of electrons, it induces an electron depletion layer at the interface with the host semiconductor SnO_2_. By reacting with TEA, CeO_2_ is induced to release electrons back into the semiconductor conduction band. Especially on the surface of CeO_2_, the redox cycle of Ce^4+^/Ce^3+^ can be realized quickly and repeatedly under certain conditions, which makes oxygen vacancies easy to produce and diffuse [31,33]. Therefore, the doping of CeO_2_ can make the gas sensing material extract more oxygen more quickly, thereby enhancing the gas sensing performance. CeO_2_-SnO_2_ sensor has high selectivity for TEA in reducing gases such as methanol, formaldehyde, acetone and methane, which may be attributed to the electron-donating effect [4,45]. However, so far, there is no clear explanation for the sensitization mechanism, further research is still needed.

## 3. Materials and Methods

### 3.1. Sample Preparation

All of the chemical reagents was analytical grade (All of the chemical reagents was provided by Aladdin, Shanghai, China) and used without further purification in experiments, including Stannous chloride dihydrate (SnCl_2_·2H_2_O), Trisodium citrate dihydrate (Na_3_C_6_H_5_O_7_·2H_2_O), Sodium hydroxide (NaOH), Cerium (III) nitrate hexahydrate (Ce(NO_3_)_3_·6H_2_O) and absolute ethanol. Deionizer water was used throughout the experiments. In a typical process of synthesize CeO_2_-SnO_2_ composites, 3.6 g SnCl_2_·2H_2_O and 11.76 g Na_3_C_6_H_5_O_7_·2H_2_O were dissolved into 40 mL of distilled water with stirring. Subsequently, 0.182 g (3 wt.%) Ce(NO_3_)_3_·6H_2_O added into the above mixture with ultrasonic treatment for 20 min. Then 40 mL of NaOH solution was dropped into the above mixture under continuous magnetic stirring. Following, the above homogenous solution was transferred to a 100 mL of stainless autoclave lined with a Teflon vessel and heated to 180 °C for 12 h. The reaction system was then cooled naturally to room temperature after reaction. This pale yellow precipitate was collected by centrifugation and washed several times with distilled water and ethyl alcohol absolute and then dried at 60 °C for 12 h. Through varying the amount of Ce(NO_3_)_3_·6H_2_O in the synthesis process, the CeO_2_-SnO_2_ composites with 0 wt.%, 3 wt.%, 5 wt.% and 7 wt.% CeO_2_ decorated SnO_2_ were prepared and denoted as SC-0, SC-3, SC-5, SC-7, respectively.

### 3.2. Characterizations

The phase and purity of the unloaded SnO_2_ and CeO_2_-doped SnO_2_ samples was investigated by powder X-ray diffraction (XRD, Bruker-AXS D8, Bruker, Madison, WI, USA) with Cu Kα radiation at 40 kV and 150 mA in a scanning range of 20–80° (2θ) and in the continuous mode with step size of 0.02° (2θ) by scanning speed of 10°/min. The morphology and nanostructure were studied by field-emission scanning electron microscopy (FESEM, Quanta™ FEG 250) (FEI, Eindhoven, The Netherlands). Chemical composition analysis was tested by energy dispersive spectroscopy (EDS, INCA ENERGY 250) (FEI, Eindhoven, The Netherlands) integrated into the FESEM system.

### 3.3. Gas Sensor Fabrication and Analysis

**Figure 7:**
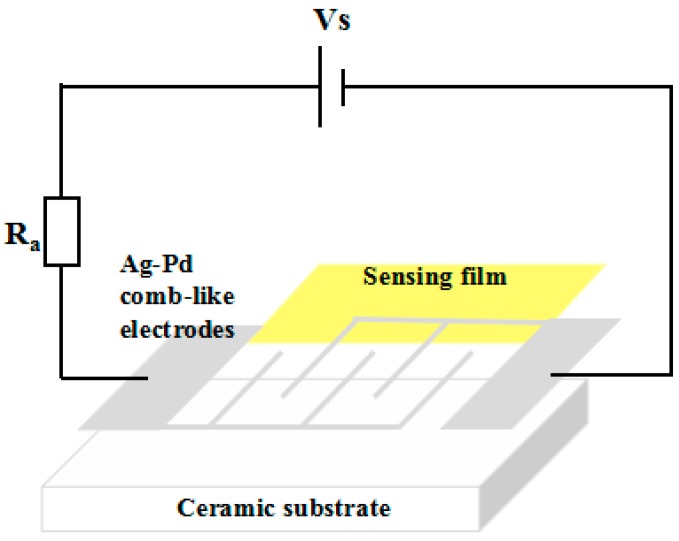
Structure diagram of gas sensor.

The sensor is prepared similarly to the method we reported previously [46,47]. In brief, the sample was mixed with distilled water to form a uniform paste and coated onto a ceramic substrate (13.4 mm × 7 mm) with an Ag-Pd electrode to obtain a resistive sensor. The structure was shown in Figure 7. In order to improve the stability and repeatability of the sensor, the gas sensitivity test was performed after aging for 12 h at 60 °C. The gas sensitivity test was conducted on the intelligent gas sensing analysis system of CGS-4TPS (Beijing Elite Tech Co., Ltd., Beijing, China) under laboratory conditions (30 RH%, 30 °C). The sensitivity of the sensor was defined as S = R_a_/R_g_, where Ra and Rg represent the resistance of the sensor in air and target gas, respectively.

## 4. Conclusions

In summary, the flower-like CeO_2_-doped SnO_2_ nanostructures were successfully synthesized by a facile one-step hydrothermal synthesis reaction using Na_3_C_6_H_5_O_7_·2H_2_O as stabilizer, SnCl_2_·2H_2_O and Ce(NO_3_)_3_·6H_2_O as precursors. Various characterizations indicate that flower-like SnO_2_ is a rutile structure with high crystallinity and CeO_2_ well modified the surface of the flower-like SnO_2_. The response of 5 wt.% CeO_2_-SnO_2_ sensor to 200 ppm TEA at the optimal working temperature of 310 °C is 252.2, which is about 3.8 times higher than that of undoped one. Moreover, SC-5 sensor displayed better selectivity for triethylamine. The improved gas-sensing performances of the composite were explained possibly due to the formation of n-n heterojunctions between CeO_2_ and SnO_2 and_ the presence of Ce^4+^/Ce^3+^ species in SnO_2_ facilitates the interaction of electrons. Therefore, the CeO_2_-doped SnO_2_ sensors can be an ideal candidate for the detection of triethylamine gas.

## Figures and Tables

**Table 1:** TEA sensing performance comparison between this study and other reported results.

Materials	TEA Concentration (ppm)	Temperature(°C)	Response(R_a_/R_g_)	Ref.
SnO_2_	200	350	5.9	[4]
Au@ZnO/SnO_2_	200	300	160	[28]
Au@SnO_2_/α-Fe_2_O_3_	200	300	63	[37]
ZnFe_2_O_4_/α-Fe_2_O_3_	200	305	65	[38]
CoMoO_4_	200	600	110	[39]
CeO_2_	100	Room temperature	4.67	[40]
Ce-doped In_2_O_3_	200	130	61.9	[41]
SC-5	200	310	252.2	this work
